# Supplementary figures and images for: Induction of zinc conjugated with Doxorubicin for the prevention of aggregating β-catenin in the Wnt signaling pathway investigated through computational approaches
Source: PLoS One. 2025 Apr 7;20(4):e0316665. doi: 10.1371/journal.pone.0316665 (PMC11975384; doi:10.1371/journal.pone.0316665)

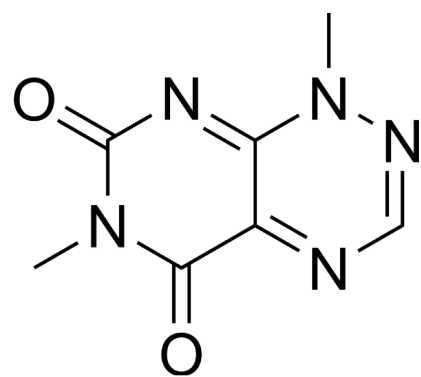

**a**

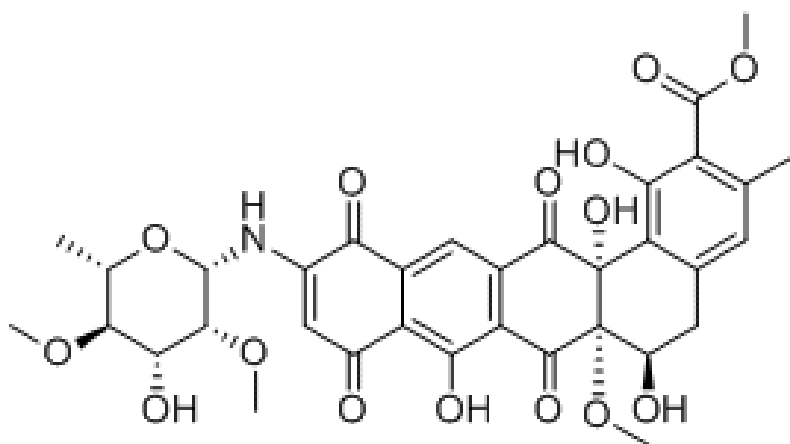

**b**

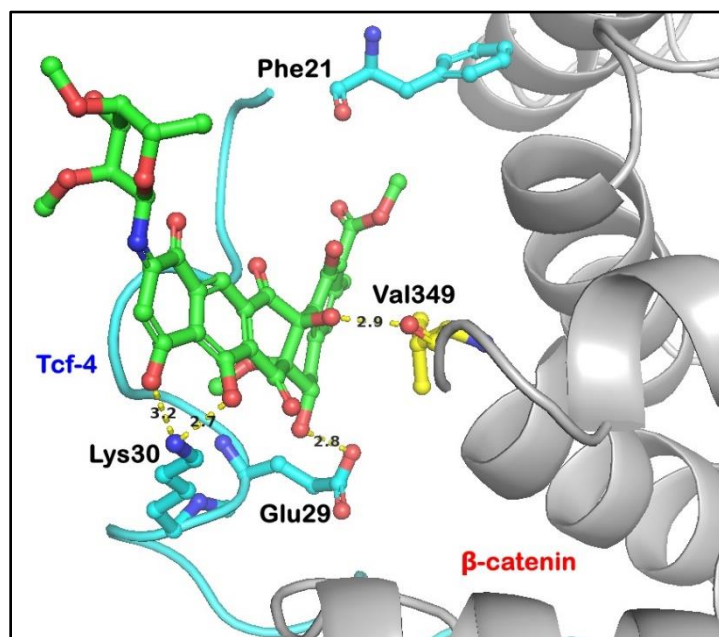

**c**

Supplement: S1 Fig — (a) Structure of PKF118-310. (b) Structure of ZTM000990. (c) Interaction between protein and ZTM000990. The backbone of Val349 alone shown interaction with the compound. Some regions of the compound are turned off from β-catenin. (PDF) [file pone.0316665.s001.pdf]

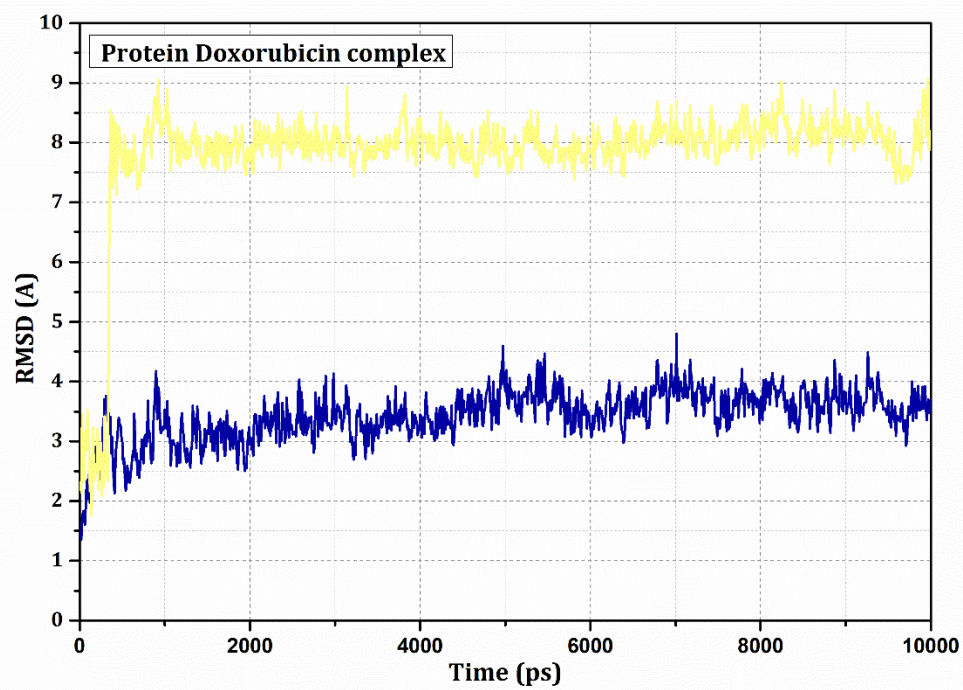

Supplement: S2 Fig — (PDF) [file pone.0316665.s002.pdf]

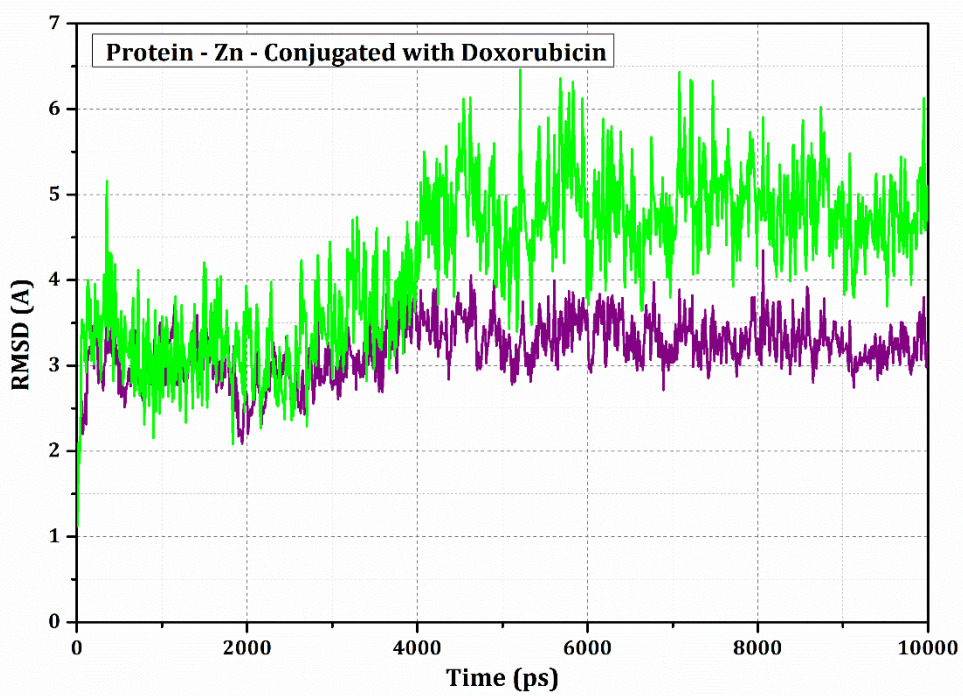

Supplement: S3 Fig — (PDF) [file pone.0316665.s003.pdf]

**RMSD Values:**

Starting Frame - 20ns : 3.06 Å

Starting Frame - 60ns : 3.30 Å

Starting Frame - 100ns : 3.76 Å

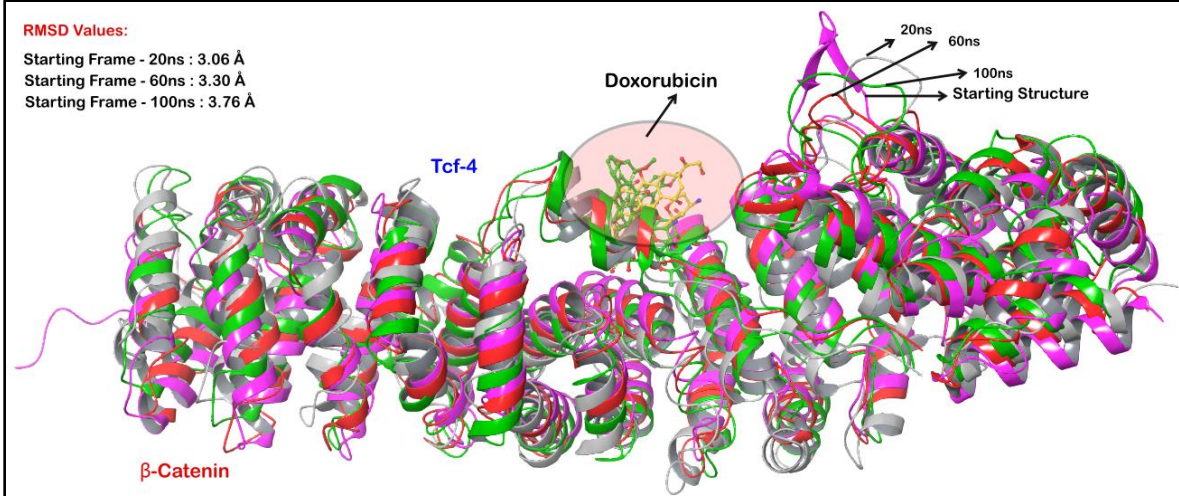

Supplement: S4 Fig — (PDF) [file pone.0316665.s004.pdf]

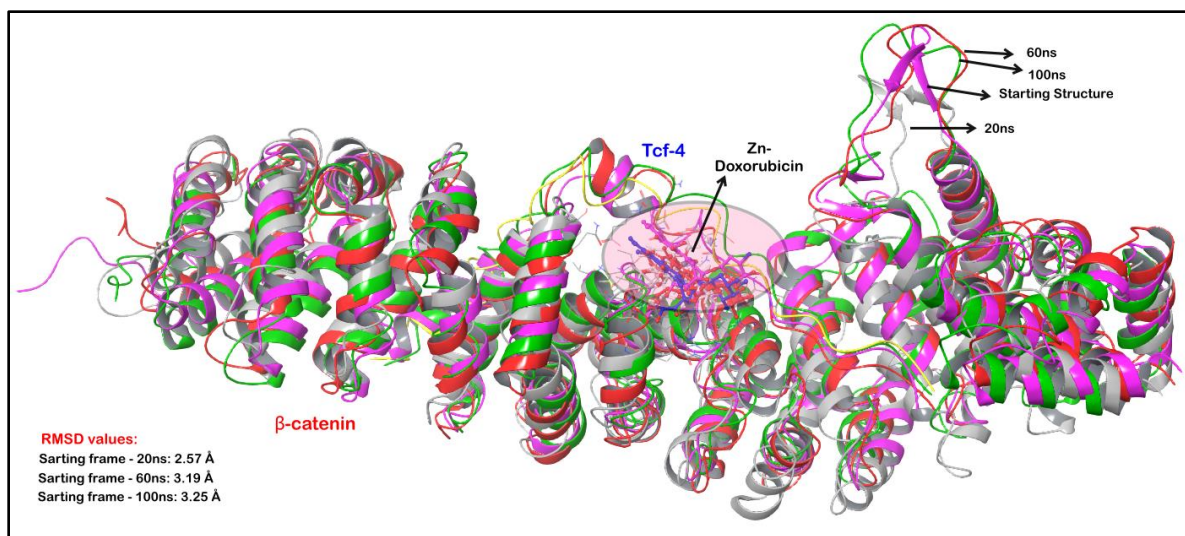

Supplement: S5 Fig — (PDF) [file pone.0316665.s005.pdf]
