# Supplementary material for: Induction of zinc conjugated with Doxorubicin for the prevention of aggregating β-catenin in the Wnt signaling pathway investigated through computational approaches
Source: PLoS One. 2025 Apr 7;20(4):e0316665. doi: 10.1371/journal.pone.0316665 (PMC11975384; doi:10.1371/journal.pone.0316665)
Supplement: S6 Fig — (PDF) [file pone.0316665.s006.pdf]

β

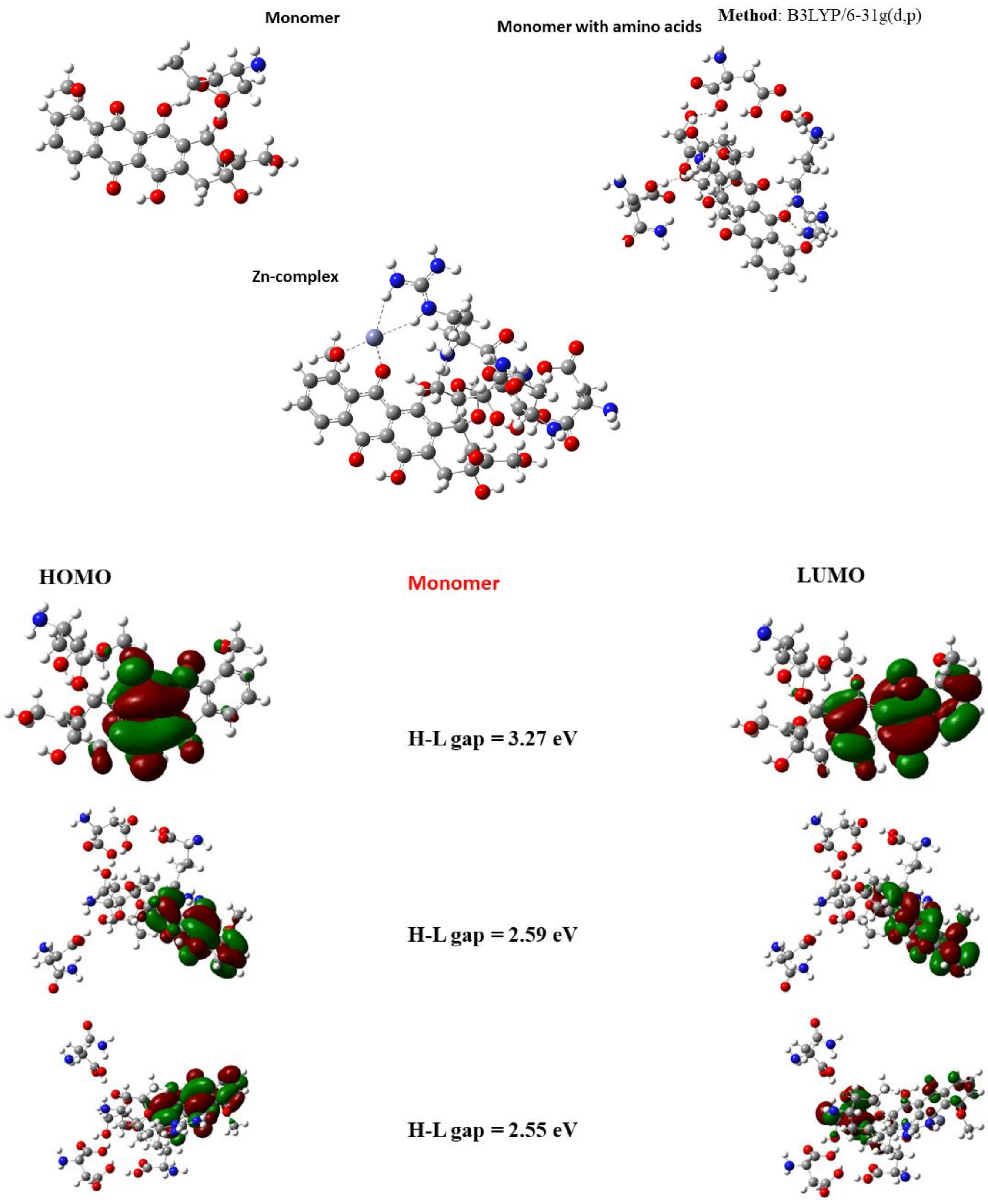

Method: B3LYP/6-31g(d,p)

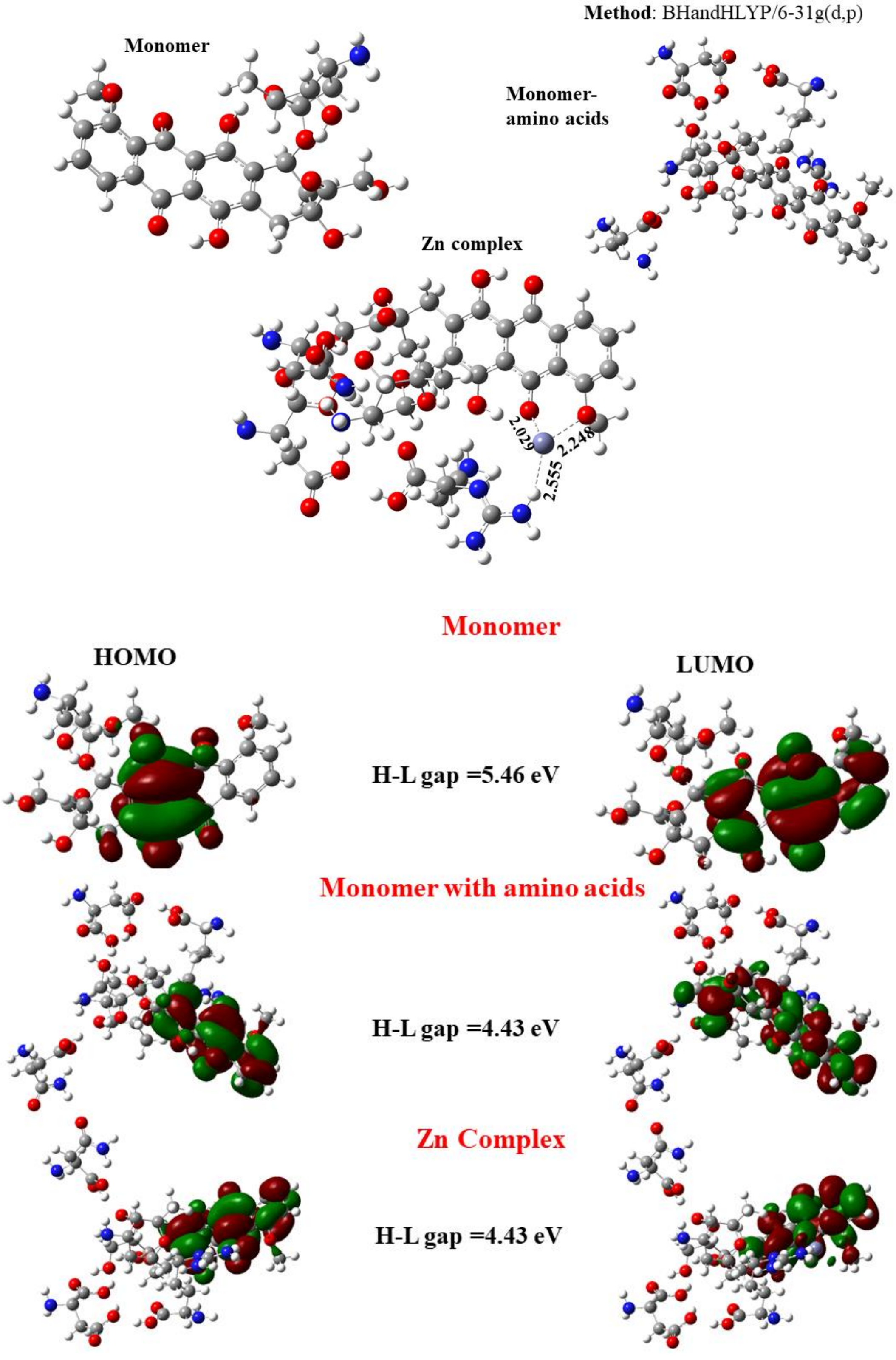

Method: BHandHLYP/6-31g(d,p)

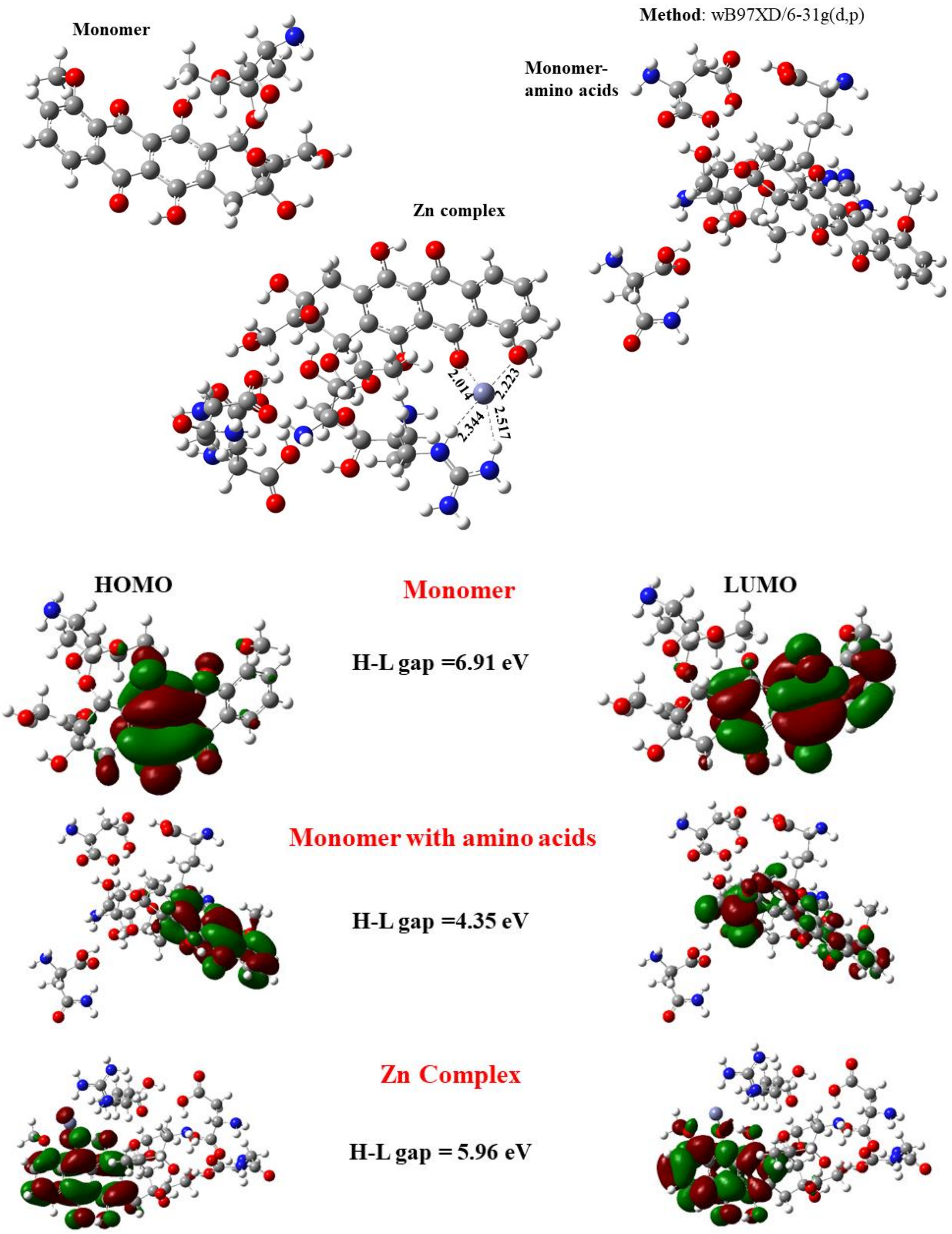

Method: wB97XD/6-31g(d,p)
