# Supplementary material for: Induction of zinc conjugated with Doxorubicin for the prevention of aggregating β-catenin in the Wnt signaling pathway investigated through computational approaches
Source: PLoS One. 2025 Apr 7;20(4):e0316665. doi: 10.1371/journal.pone.0316665 (PMC11975384; doi:10.1371/journal.pone.0316665)
Supplement: Attachment — Submitted filename: Response_to_Reviewers.docx [file pone.0316665.s008.docx]

**Response to Reviewers**

**Reviewer #1:**

Recommendation: Publish after major revisions noted.

The manuscript by Gebru and co-workers explored the role of free Doxorubicin and zinc conjugated Doxorubicin against β-catenin in the treatment of cervical cancer. The study has been conducted purely from the computational standpoint where the authors have used Molecular Docking, Molecular Dynamics and Density Functional Theory (DFT) to understand the stabilizing factors present in both cases. While the manuscript furnishes some insightful data, I found the manuscript to be poorly written, difficult to follow with many inconsistencies. The manuscript may be published after rewriting it in a more compact manner. The abstract is also filled with redundant information and may be shortened. There are certain additional comments which are listed below:

1. **The authors have carried out the DFT studies in a number of functionals. It is quite impressive. However, I would like to see the numbers in B3lYP-D3 as I think dispersion correction will play a significant role in these systems.**

**Response:** We have calculated the binding energy at B3LYP-D3 level of theory in the gas phase and the results were added in the manuscript. The results of binding energy at B3LYP-D3 optimization are close agreement with M06 level of theory (See Table 1). However, after the addition of metal atom the binding energy result is close in agreement with the BHandHLYP and ωB97XD level of theory. It shows that the dispersion corrected method calculates the binding energy of metal complexes reasonably good and given in Page Number: 15

2. **Have the authors considered QM/MM studies? I am curious as to what happens when the structures are subjected to the entire protein environment.**

**Response:** Thanks for your comments.

Due to the limitation of software availability, we will execute the QM/MM studies in the future. However, the structure in the protein environment is addressed by Molecular dynamics simulation and the results are shown in MD simulation

**Reviewer #2:** The overall paper is a very interesting read and is very well put together. Here are a few comments and clarifications:

1. **First part of paragraph 1 lacks references (eg: line 3, line 4)**

**Response:** Thanks for your comments. We have inserted the reference in the line 3 and 4 and shown in Page Number: 2

2. **Page 5 paragraph 2: Reference missing for FDA claim and missing references in a fewer line after that**

**Response:** Thanks for your comments. We have included the references in that section and given in the page number: 5.

3. **Page 6: ChemDraw and Schrodinger have not been referenced**

**Response:** Thanks for your comments. The appropriate reference has been cited for Chemdraw and Schrodinger, it was shown in the Page Number: 6.

4. **On what basis was the Zinc conjugated to the doxorubicin? Was it mostly chemical intuition or is there previous studies that were done for it?**

**Response:** Thanks for your comments.

Gewirtz D. A. (1999). A critical evaluation of the mechanisms of action proposed for the antitumor effects of the anthracycline antibiotics adriamycin and daunorubicin. Biochemical pharmacology, 57(7), 727–741. https://doi.org/10.1016/s0006-2952(98)00307-4 propsed mechanisms by which doxorubicin acts in the cancer cell (i) intercalation into DNA and disruption of topoisomerase-II-mediated DNA repair and (ii) generation of free radicals and their damage to cellular membranes, DNA and proteins.

Zinc is essential for microorganisms, plants, and animals. Deprivation of zinc arrests growth and development and produces system dysfunction in these organisms. Zinc provides the structural integrity of proteins and the regulation of gene expression. Hence, we have conjugated the Zinc with Doxorubicin to understand insights to analyse how the Zn-drug conjugates can effectively target malignant cells and induce apoptosis.

5. **How were the missing residues added to the PDB?**

**Response:** Thanks for your comments.

The missing residues were added when preparing the protein using Protein Preparation Wizard of Schrodinger and shown in Page Number: 6

6. **What are the forcefields used for MD simulations?**

**Response:** Thanks for your comments.

The Molecular Dynamics Simulation was performed Desmond package with OPLS2005 force field and shown in Page Number:7.

7. **Is 50 ns enough simulation time? Were there replicates done for the simulation?**

**Response:** Thanks for your comments.

The Molecular Dynamics Simulation was performed for the period of 100ns as triplicates. The average interaction and their contributions are given in Figure 6 and 7 and it was shown in Page Number: 11 to 13.

8. **What were the basis of choosing the specific model chemistries?**

**Response:** Thanks for your comments

The chosen model of drug loading chemistry effectively combines enhanced stability, high loading efficiency, and controlled release, while enabling targeted delivery and maintaining biocompatibility. This comprehensive approach is designed to improve the efficacy of cancer therapies, optimize patient outcomes, and address the challenges associated with conventional drug delivery systems.

9. **Page 8: Missing reference for STRING Database**

**Response:** Thanks for your comments.

The reference for the STRING database were added and shown in Page Number: 8

10. **Page 13 and 14: Figure 7 can be separated into 2 figs or can be made smaller to accommodate in one page**

**Response:** Thanks for your comments

The Figures are renumbered. Hence, the figures are updated as Figure 6 and Figure 7 and shown in Page Number: 12. The RMSD figures of the Molecular Dynamics simulation are moved to supplementary material and numbered as S1 and S2.
