# Supplementary material for: Induction of zinc conjugated with Doxorubicin for the prevention of aggregating β-catenin in the Wnt signaling pathway investigated through computational approaches
Source: PLoS One. 2025 Apr 7;20(4):e0316665. doi: 10.1371/journal.pone.0316665 (PMC11975384; doi:10.1371/journal.pone.0316665)
Supplement: Attachment — Submitted filename: Reviewer_coments.docx [file pone.0316665.s009.docx]

**Reviewer Comments**

Reviewer #3: Authors of the presented manuscript evaluated the computational affinity Doxorubicin and Zinc conjugated with Doxorubicin against β-catenin biotarget for identifying promising anti-cancer agents. Study is relevant to its field. Following comments and suggestions should be addressed:

1. Within docking study, authors should elaborate more on the polar hydrogen bond interactions. Hydrogen binding should further be presented within hydrogen bond distances as well as hydrogen bond angles since hydrogen bond depends on both. Authors should mention the Hydrogen bond angles as well as their distances since the strength of hydrogen bonding is based on both parameters in a way to ensure the adequacy of optimum hydrogen bonding.

Response: Thanks for your comments.

We have displayed the hydrogen bond interactions and highlighted the distance of the complex and given in the page number: 9,10, Table -1 and shown in Fig4, 5.

2. Authors should adopt positive controls or reported reference compounds for the β-catenin target throughout the in silico studies in a way to validate and ensure the successful biological translation of the computational findings.

Response: Thanks for your comments.

Yang et al., 2021 has comprehensively listed the β-catenin inhibitors along with their experimental values in their review. We have started the screening procedure with the two β-catenin inhibitors such as (i) PKF118-310 (IC_50_: 0.8 µM) and (ii) ZTM000990 (IC_50_: 0.64 µM) as a reference. The PKF118-310 has shown the binding energy -4.13 kcal/mol and ZTM000990 has shown -8.50 kcal/mol (~0.642 μM) (Figure S1). Hence, ZTM000990 is taken as a reference compound for the present study and given in Page Number: 11.

3. At molecular dynamics study, authors should explore the comparative mm-PBSA/GBSA free binding energies for each ligand-target complex to highlight the nature (electrostatic, van der Waal, and solvation) and magnitude of binding the thing that would guide further compound’s development and optimization.

Response: Thanks for your comments

**MM/GBSA analysis**

The Binding Free Energy was computed every 20^th^ frame of the protein-ligand complex, and the average value was reported. The ZTM000990 has shown an average value of -52.84 kcal/mol followed that the Doxorubicin and Zn-Doxorubicin resulted in -48.25 kcal/mol, -57.36 kcal/mol, respectively. The study suggests that Zn-Doxorubicin has shown better interaction with the Val349, Lys354, and Arg386; Phe21 of Tcf-4. In contrast, the Doxorubicin has shown interaction with charged residues and less interaction with Phe21 (hydrophobic residue). The dG_coulomb_ for ZTM00990, Zn-Doxorubicin and Doxorubicin has shown -39.26, -68.93 and -35.66 kcal/mol respectively (Table – 2). This study shows that interaction energy between the protein and ligand is found optimized in the presence of Zn^2+^ and due to the presence of hydrophobic residues in the binding site, hence, the complex was identified as stable. The lowest energy of ΔG_bind_ values indicates the compactness of the ligands inside the binding pocket of β-catenin.

Solvation and desolvation effects are considered a most effective approach to evaluate the global and local conformation of protein in the solvent model[46]. The solvation-free energy values were calculated for every residue, and it was averaged. The averaged solvation energy for ZTM-000990 has shown -5.35 kcal/mol, and Doxorubicin and Zn-Doxorubicin complex has shown -3.38 and -6.55 kcal/mol respectively. The difference between the Zn-Doxorubicin and ZTM-000990 has shown ~1.0 kcal/mol order of magnitude whereas the Doxorubicin has shown ~ 3 kcal/mol. However, the Zn^2+^ has a role of altering the conformation as well as attracting possible contacts to maintain stability and shown in page number: 16

4. Further, authors are advised to provide overlays for the initial, middle, and final frames (at 0 ns, 50 ns, and 100 ns, respectively) for each ligand-protein complex across the molecular dynamics simulation. This approach would provide great insights regarding the time-evolution orientation/conformation changes for both the protein and bounded ligands as well as the conserved and reformed ligand-amino acid bindings and close-range contacts.

Response: Thanks for your comments

We have extracted the structures of every 20ns, it has been overlaid with the starting structure. The RMSD values were observed for the same shown in Figure S3 and S4. The conformational changes occurred ~ 1 Å from the starting to end frames. Further, the Tcf-4 has changed a bit during simulation shown in Page number: 20 (Figure 11) and shown in Supplementary Figure S4, S5.
